# Lipid mediator profile in vernix caseosa reflects skin barrier development

**DOI:** 10.1038/srep15740

**Published:** 2015-11-02

**Authors:** Antonio Checa, Tina Holm, Marcus O. D. Sjödin, Stacey N. Reinke, Johan Alm, Annika Scheynius, Craig E. Wheelock

**Affiliations:** 1 Division of Physiological Chemistry 2, Department of Medical Biochemistry and Biophysics, Karolinska Institutet, Stockholm, Sweden; 2 Translational Immunology Unit, Department of Medicine Solna, Karolinska Institutet and University Hospital, Stockholm, Sweden; 3 Department of Clinical Science and Education, Södersjukhuset, Karolinska Institutet and Sachs´ Children and Youth Hospital, Stockholm, Sweden

## Abstract

Vernix caseosa (VC) is a protective layer that covers the skin of most human newborns. This study characterized the VC lipid mediator profile, and examined its relationship to gestational period, gender of the newborn and maternal lifestyle. VC collected at birth from 156 newborns within the ALADDIN birth cohort was analyzed and 3 different groups of lipid mediators (eicosanoids and related oxylipin analogs, endocannabinoids and sphingolipids) were screened using LC-MS/MS. A total of 54 compounds were detected in VC. A number of associations between lipid mediators and the gestational period were observed, including increases in the ceramide to sphingomyelin ratio as well as the endocannabinoids anandamide and 2-arachidonoylglycerol. Gender-specific differences in lipid mediator levels were observed for all 3 lipid classes. In addition, levels of the linoleic acid oxidation products 9(10)-epoxy-12Z-octadecenoic and 12(13)-epoxy-9Z-octadecenoic acid (EpOMEs) as well as 12,13-dihydroxy-9Z-octadecenoic acid (DiHOME) were increased in VC of children from mothers with an anthroposophic lifestyle. Accordingly, VC was found to be rich in multiple classes of bioactive lipid mediators, which evidence lifestyle, gender and gestational week dependencies. Levels of lipid mediators in VC may therefore be useful as early stage non-invasive markers of the development of the skin as a protective barrier.

Vernix caseosa (VC) is a cheesy layer that covers the skin of the human fetus during the last trimester of pregnancy and is usually present on the skin of full-term newborns. It is a mixture of water (~80%), proteins (~10%) and lipids (~10%)^1^. VC has a protective function, forming an innate defense barrier that protects the fetus and the newborn from external insult^2^. Due to its direct contact with skin early in life, VC has been hypothesized as a facilitator of the formation of the stratum corneum (SC)^3^, the outermost layer of the skin. Thus, the composition of VC has been suggested as a non-invasive surrogate of fetal skin synthesis and maturation^1^. Therefore, VC may be a useful matrix to address the question of the status of maturity of the skin as a protective barrier at birth.

Because of the increasing interest in their influence in skin inflammation and immunity^4^, lipid mediators have been studied in multiple biofluids and tissues, including VC. Ceramides in particular have been investigated in VC due to their role in the epidermis. A relationship between the VC ceramide pattern and mid-gestational fetal and neonatal epidermis has been reported^1^. However, the presence of other sphingolipids as well as other bioactive lipid mediators such as the oxygenated products of polyunsaturated fatty acids, including eicosanoids and related analogs (oxylipins) and the endocannabinoids has not been described in VC. These compounds are known to be present in the skin^4^ and to be altered in pathological conditions such as skin inflammation^5^. Interestingly, a recent study showing gender differences in the lipid fraction of VC suggested the possibility of a dynamic composition of VC with the number of gestational weeks^6^. Therefore in the present study we aimed to determine the presence of lipid mediators in VC and to elucidate if these two factors, gender and gestational week could also affect their composition in VC. This analysis was performed by using samples from the Assessment of Lifestyle and Allergic Disease During INfancy (ALADDIN) birth cohort^7^, which enabled us to also inspect the influence of the lifestyle of the mother in the composition of VC.

## Results

### Study population

Demographic data from the 156 children presented in Table 1 do not show any significant differences in terms of gestational week, gender of the children and lifestyle of the mother for any of the different experimental designs. The differences between the three lifestyles are presented in Table 2. The diet of the mothers during pregnancy differed significantly between the lifestyle groups. The anthroposophic mothers were vegetarians to a higher degree than the partly- or non-anthroposophic mothers and organic/biodynamic diet was more common in the anthroposophic group than in the partly- or non-anthroposophic group. Using butter on bread and when cooking was more common in the anthroposophic lifestyle group than in the other two groups (Table 2).

### Lipid mediators in VC

Of the 133 lipid mediators screened in the 156 VC samples, 80 (43 oxylipins, 23 sphingolipids and 14 endocannabinoids) were present above the limit of detection in ≥50% of the samples. A total of 54 lipid mediators (21 oxylipins, 23 sphingolipids and 10 endocannabinoids) presented a coefficient of variation (CV) <30% in the pooled QC samples and were present in ≥70% of the samples. The concentrations of these 54 lipid mediators, included in further analyses, are reported in Supplementary Table S1.

### Sphingolipid and endocannabinoid levels in VC are affected by the gestational period

**Figure 1:**
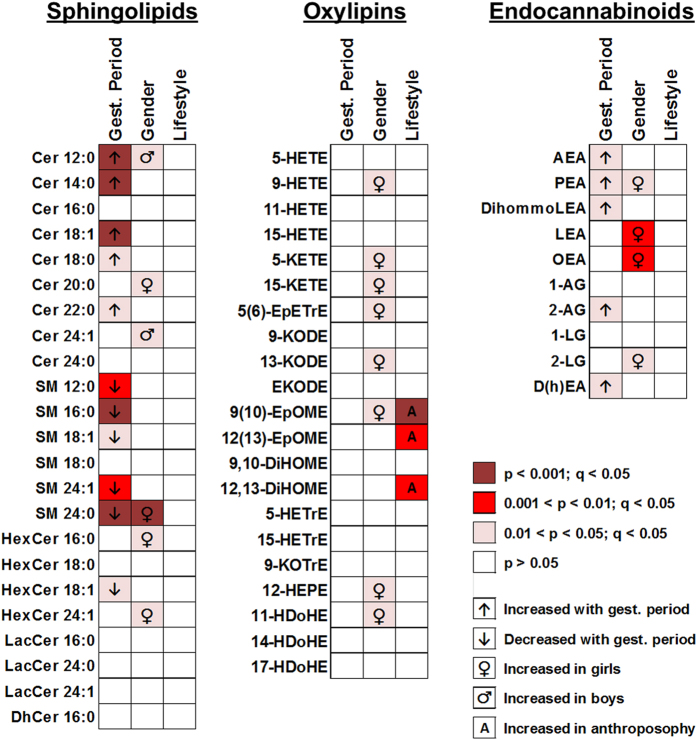
Levels of sphingolipid, oxylipin and endocannabinoid species in vernix caseosa (VC) affected by gestational period, gender of the child or lifestyle of the mother. See Supplementary Table S1 for compound nomenclature. P-values for the linear regression of one of the factors adjusted for the other two as covariates are displayed. All indicated changes were corrected for multiple hypothesis testing using the Storey q-value.

**Figure 2:**
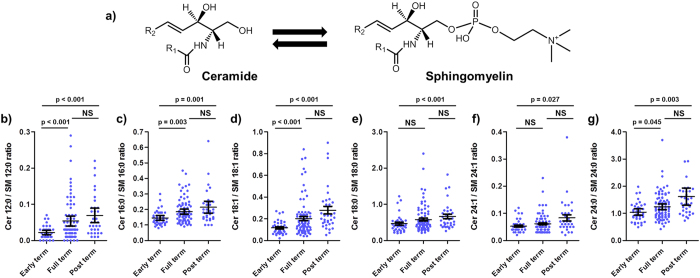
The ceramide to sphingomyelin ratio increased with gestational period. (**a**) General structure for ceramides and sphingomyelins belonging to the non hydroxylated fatty acid/sphingosine backbone (NS) sphingolipid class. (R1 = fatty acid alkyl chain; R2 = (CH_2_)_12_CH_3_)). (**b**–**g**) levels for specific R1 chain lengths according to the gestational period. Early term = 36–38 weeks, Full term = 39–40 weeks and post term = 41–42 weeks. Bonferroni corrected p-values corresponding to the linear regression of gestational period adjusted for lifestyle of the mother and gender of the baby are presented. Each point represents an individual. The arithmetic mean with 95% confidence intervals is presented.

A series of lipid mediators, mainly ceramides, sphingomyelins and endocannabinoids and related molecules showed dependence with the gestational period. In particular, a general increase in ceramides with a concomitant decrease in sphingomyelin levels was observed (Fig. 1). As ceramides and sphingomyelins can be enzymatically interconverted (Fig. 2a), the ceramide/sphingomyelin ratio was also inspected for all compounds where both chain lengths were available. Results for the regression of all chain lengths showed a significant increase with gestational period after adjusting for gender and lifestyle: Cer/SM_12:0_ (p < 0.001), Cer/SM_16:0_ (p < 0.001), Cer/SM_18:1_ (p < 0.001), Cer/SM_18:0_ (p = 0.006), Cer/SM_24:0_ (p < 0.001) and Cer/SM_24:1_ (p = 0.008). Differences for all gestational periods are presented in Fig. 2b–g. In addition, levels of the endocannabinoids anandamide (AEA) and 2-arachidonoyl glycerol (2-AG), correlated with the ceramide/sphingomyelin ratio for all compounds except for AEA and Cer/SM_24:0_ (Supplementary Table S2).

### Boys and girls exhibit different VC lipid mediator profiles

Gender related differences were found for lipid mediators belonging to the three lipid mediator families included in the study (Fig. 1). Specifically, increased levels of some oxylipins were observed in girls relative to boys. No differences were observed for the ceramide to sphingomyelin (Cer/SM) ratio in boys relative to girls: Cer/SM_12:0_ (p = 0.311), Cer/SM_16:0_ (p = 0.560), Cer/SM_18:1_ (p = 0.602), Cer/SM_18:0_ (p = 0.386), Cer/SM_24:0_ (p = 0.251) and Cer/SM_24:1_ (p = 0.054).

### Linoleic acid-derived compounds shift on a lifestyle basis

**Figure 3:**
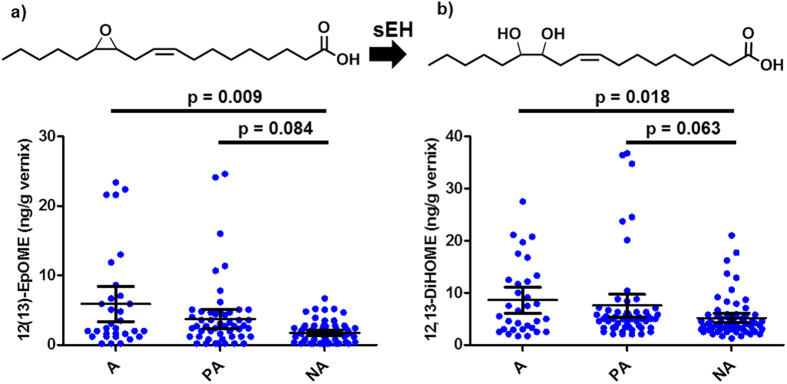
Structures and phenotypic differences in levels of (a) 12(13)-epoxy-9Z-octadecenoic acid (12[13]-EpOME) and (b) 12,13-dihydroxy-9Z-octadecenoic acid (12,13-DiHOME) according to lifestyle (A = anthroposophic; P = partly anthroposophic; NA = non- anthroposophic). The 12(13)-EpOME is converted to the 12,13-DiHOME via activity of the enzyme soluble epoxide hydrolase (sEH). Bonferroni corrected p-values corresponding to the linear regression of lifestyle of the mother adjusted for gestational period and gender of the baby are presented. Each point represents an individual. The arithmetic mean with 95% confidence intervals is presented.

No differences were found in sphingolipids or endocannabinoids in relation to the maternal lifestyle (Fig. 1). However, linoleic acid-derived oxylipins formed from the cytochrome P450 pathway significantly increased in the order non-anthroposophic < partly anthroposophic < anthroposophic lifestyle after adjusting for gestational period and gender of the child (Fig. 1): 9(10)-epoxy-12Z-octadecenoic acid (9[10]-EpOME; p = 0.001; q = 0.008), 12(13)-epoxy-9Z-octadecenoic acid (12[13]-EpOME; p = 0.002; q = 0.008), and 12,13-dihydroxy-9Z-octadecenoic acid (12,13-DiHOME; p = 0.002; q = 0.008). The example of 12(13)-EpOME and 12,13-DiHOME is shown in Fig. 3. The observed increase with anthroposophic lifestyle in the other linoleic acid-derived regioisomer from the same pathway, 9,10-dihydroxy-12Z-octadecenoic acid (9,10-DiHOME) did not reach significance after adjusting for multiple comparisons (Supplementary Figure S1a; p = 0.03; q = 0.07). No associations were observed for the three other linoleic acid-derived oxylipins with lifestyle (Fig. 1, Supplementary Figure S1b–d).

## Discussion

Multiple lipid mediators are known to be important in skin health either as structural components (*e.g.*, ceramides) or as having a key role in skin inflammation and immune regulation^4^. Accordingly, determining the skin lipid mediator composition at birth may provide insights into the integrity and functionality of this barrier in the newborn. However, given the ethical issues associated with the invasiveness of sample collection in newborns (*i.e.,* skin biopsies)^8^, skin functionality is usually assessed by the measurement of other surrogates such as the transepidermal water loss^9^.

In the present study, we inspected the feasibility of VC to provide information related to skin maturity by quantifying a series of lipid mediators belonging to the sphingolipid, endocannabinoid and oxylipin families. Lipid mediator levels were observed to associate with the gestational period, gender of the newborn and the lifestyle of the mother. Previous works reporting the presence of ceramides^1^ and other lipids^6^ in VC suggested the possibility that VC may reflect fetal epidermal synthesis. In addition, ceramides along with other epidermal lipids, have been previously described in VC and their pattern mirrors that of mid-gestational and early postnatal epidermis^1^. Due to their role in skin barrier function, ceramides are associated with a number of skin diseases^4^ and are being promoted for therapeutic use in skin care products^10^. VC contains the same ceramide classes as stratum corneum and in similar proportions^11^. We analyzed sphingolipids belonging to the ceramide NS class (*i.e.*, non hydroxylated fatty acid/sphingosine backbone), one of the most abundant in VC^1^,^11^ and one of the two sphingolipid families where ceramides can be derived from sphingomyelins in the stratum corneum^12^. Our observation of increases in the ceramide/sphingomyelin ratio with gestational age (Fig. 2b–g) supports the theory that VC may be used as a surrogate of fetal epidermis synthesis^1^. This may be a reflection of the cornification process in the stratum corneum formation of the fetus, where increased levels of ceramides are required to assist the newborn in adapting to the dry environment^13^. Sphingomyelins are the likely NS-ceramide source^12^, as evidenced by their concomitant decrease with increasing ceramide levels. Interestingly, although epidermal barrier lipid synthesis has been found to be delayed by androgens in rats^14^, we observed no differences in the dynamics of the ceramide/sphingomyelin ratio with the gestational period in boys relative to girls. However, one study did report decreased epidermal barrier lipids in VC from males^15^, findings which could not be corroborated in follow-up studies^1^. In the current investigation, although mild differences were observed for some sphingolipid species depending on the gender of the newborn (Fig. 1), no changes were observed in the ceramide/sphingomyelin ratios. The current work focused only on the NS class of sphingolipids, and gender may of course still exert influence on other families of sphingolipids. Specifically, it would be interesting to examine possible alterations in sphingolipids containing an esterified omega-hydroxy fatty acid (EO), because they are known to be especially relevant in maintaining epidermal barrier and function^4^.

Endocannabinoids are endogenous lipid mediators that target the cannabinoid receptors. They are known to be involved in the regulation of skin physiology^16^, and play an important role during pregnancy^17^. In the present study, we report for the first time the presence of a series of endocannabinoids and related molecules, including the two prototypical endocannabinoid molecules, AEA and 2-AG in VC. The fetus is exposed to changes in AEA levels *in utero* depending on the gestational period, with initial lower levels that increase as term approaches^18^. Our data show that changes of AEA and 2-AG can be observed in VC even within the short time span (36 to 42 weeks) of its collection relative to the total time of gestation (Fig. 1). In addition, levels of AEA (and also of 2-AG) positively correlated with all ceramide/sphingomyelin ratios (except in the case of AEA and ceramide/sphingomyelin 24:0). AEA has been shown to inhibit epidermal differentiation in keratinocytes^19^, and thus increasing levels with gestational period may be related to the ongoing cornification process reflected by the increased ceramide to sphingomyelin ratio. Moreover, the activation of cannabinoid receptors has been shown to induce ceramide generation by activation of sphingomyelinases in astrocytes^20^. Thus, VC arises as an additional non-invasively collected source for the study of endocannabinoids and their interaction with other lipid mediators early in life.

Finally, the use of samples belonging to the ALADDIN cohort^7^ enabled us to study the influence of anthroposophic lifestyle on the composition of VC. Anthroposophy involves a lifestyle that tends towards home deliveries, a vegetarian diet, and restricted use of antibiotics as well as several other features reported elsewhere^7^. In the present study, only differences in home deliveries and diet were observed (Table 2). A number of linoleic acid-derived oxidation products were found to increase with the mother’s degree of anthroposophic lifestyle (Figs 1 and 3). These compounds are joint products of cytochrome P450 and soluble epoxide hydrolase (sEH) activity (EpOMEs and DiHOMEs, respectively)^21^. In the anthroposophic lifestyle group there was an increased use of butter both on bread and relative to vegetable oils when cooking as well as a higher prevalence of vegetarian diet (Table 2). Accordingly, the contribution of diet to the fatty acid content of the VC is unclear, with a potential shift in saturated (*e.g.,* stearic acid) as well as unsaturated fatty acid (*e.g.,* linoleic acid) species. There is unfortunately little information in the literature on the levels of fatty acids in VC and future studies would clearly benefit from quantification of the fatty acid composition of VC. This information would in particular aid in interpreting the effect of diet on the biochemical pathways responsible for lipid mediator production.

It is also plausible that the observed lipid mediator changes reflect differential activities of the enzymes involved in metabolite biosynthesis rather than shifts in the levels of the fatty acid substrates. This observation is supported by the fact that no lifestyle-associated differences were observed for either the linoleic acid-derived potential downstream lipoxygenase products 9- and 13-KODE or the autooxidation product EKODE (Fig. 1, Supplementary Figure S1c,d). However, the activity of cytochrome P450s on arachidonic acid metabolites could not be assessed because the majority of the corresponding arachidonic acid epoxide and diol analogs (*i.e.,* the epoxyeicosatrienoic and dihydroxyeicosatrienoic acids; EpETrEs and DiHETrEs) were either not detected or did not pass the quality criteria (with the exception of 5(6)-EpETrE, which did not evidence a lifestyle-associated shift; Fig. 1). The lower levels of arachidonic acid metabolites relative to linoleic acid metabolites can be explained by the relatively higher abundance of linoleic acid in the epidermis^22^. No shifts were observed in the linoleic acid-derived linoleoyl ethanolamide (LEA) or linoleoyl glycerols (1- and 2-LG); however, this can be explained by a higher prevalence of linoleic acid incorporation into the sn-2 position of phospholipids (leading to the eicosanoid biosynthetic pathway). These results suggest that lifestyle-based differences may result in specific fluctuations in cytochrome P450-mediated linoleic acid metabolism, the implications of which should be explored in more detail.

In addition, gender-specific effects in cytochrome P450 and sEH activity have been reported, especially in relation to hypertension and cardiovascular disease^23^. In the current study, weak gender differences were observed in the cytochrome P450-derived 9(10)-EpOME and 5(6)-EpETrE (Fig. 1). Accordingly, future studies should take into account the influence of gender on the production of oxylipins and the associated subsequent potential pathobiological effects.

To summarize, the present study reports for the first time the presence of a series of bioactive lipid mediators in VC. The total gestational period, gender of the baby and lifestyle of the mother were found to differently affect the lipid mediator composition of VC. A number of potentially interesting associations were observed regarding their involvement in skin barrier development. Accordingly, levels and speciation of lipid mediators in VC may potentially be useful as early stage markers of the development of skin. The attractiveness of this matrix as a source of biomarkers is enhanced by the easiness and lack of invasion required for its collection.

## Methods

### Study population and vernix caseosa collection

Samples were collected from the birth cohort ALADDIN^7^. Inclusion criteria of VC samples in the current study required that sufficient amount of VC (15 mg) was available for lipid mediator quantification. VC samples from 156 newborns fulfilled this criterion. Demographic data are presented in Tables 1 and 2. Children born at 36–38, 39–40 and 41–42 gestational weeks were classified as pre-term, full-term and post-term, respectively (Table 1). Lifestyle classification of the mother as anthroposophic, partly anthroposophic and non-anthroposophic is described in detail in^7^ and presented for the current study in Table 2. VC from newborns was collected by midwives and stored at –80 °C until analysis. The study was approved by the Regional Ethical Review Board in Stockholm and conducted according to the Declaration of Helsinki’s principles. All parents have given their written informed consent.

### Extraction protocol

A modified Bligh and Dyer protocol^24^ was used for the extraction of lipid mediators from VC. Briefly 10 μl of internal standard mix for all 3 analytical platforms were added to 15 mg of VC and dissolved in 0.19 mL of CHCl_3_. Samples were then vortexed for 30 sec and sonicated in an ultrasound bath for 10 min. Afterwards 0.38/0.15/0.19/0.19 mL of MeOH/H_2_O/CHCl_3_/H_2_O were sequentially added with 30 sec vortex for each step. Samples were centrifuged at 3000 rcf for 5 min and the organic phase was withdrawn. VC was re-extracted with 0.30 mL of CHCl_3_ and organic extracts were combined, concentrated under vacuum, resuspended in 100 μl of MeOH and filtered using 0.1 μm spin filters (Merck Millipore, Billerica, MA, USA) before analyzing via the lipid mediator platforms described below. Six vernix quality controls (QC) were pooled from two children not included in the study and used to control for quantification reproducibility and batch extraction effects.

### Lipid mediator quantification

All lipid mediator analyses were carried out using Ultra Performance Liquid Chromatography - tandem mass spectrometry (LC-MS/MS) on an ACQUITY UPLC System from Waters Corporation (Milford, MA, USA) equipped with an autosampler cooled to 5 °C. Detection was performed using a Waters Xevo® TQ triple quadrupole (sphingolipids) or TQS triple quadrupole (TQS) (oxylipins and endocannabinoids), both equipped with an Electrospray Ion Source (ESI).

Sphingolipids were purchased from Avanti Polar Lipids (Alabaster, AL, USA), except for lactosylceramide 17:0, which was purchased from Larodan Fine Chemicals (Malmö, Sweden). Sphingolipid separation was performed using an ACQUITY UPLC BEH (Ethylene Bridged Hybrid) C8 Column (130 Å, 1.7 μm, 2.1 mm × 150 mm), equipped with a pre-column (ACQUITY UPLC BEH C8 VanGuard Pre-column, 130 Å, 1.7 μm, 2.1 mm × 5 mm), both from Waters (Milford, US). Aqueous and reverse phase mobile phases consisted of 5 mM ammonium formate/0.2% formic acid in water and methanol, respectively. The chromatographic and mass spectrometry experimental parameters were set-up as previously published^25^. Compound-specific mass spectrometric and chromatographic parameters are summarized in Supplementary Table S3.

Endocannabinoids, oxylipins and their respective internal standards were purchased from Cayman (Ann Arbor, MI, USA). Endocannabinoid and oxylipin separation was performed using an ACQUITY UPLC BEH (Ethylene Bridged Hybrid) C18 Column (130 Å, 1.7 μm, 2.1 mm × 150 mm) equipped with a pre-column (ACQUITY UPLC BEH C18 VanGuard Pre-column, 130 Å, 1.7 μm, 2.1 mm × 5 mm) (Milford, US). For endocannabinoids, the chromatographic gradient (Mobile phase A = mobile phase A = 0.1% acetic acid in water; mobile phase B = Acetonitrile/Isopropanol (90:10, v-v) was as follows: 0 min, 65% B; time range 0 → 3.0 min, 65% B (constant); time range 3.0 → 3.1 min, 65 → 80% B (linear increase); time range 3.1 → 5.0 min, 80% B (isocratic range); time range 5.0 → 5.1 min, 80 → 90% B (linear increase); time range 5.1 → 6.0 min, 90% B (isocratic range); time range 6.0 → 6.1 min, 90 → 100% B (linear increase); time range 6.1 → 9.0 min, 100% B (isocratic range); time range 9.0 → 9.1 min, 100 → 65% B (linear decrease); time range 9.1 → 11.0 min, 65% B (isocratic column conditioning). The general MS parameters were set as follows: Desolvation temperature: 550 °C; Capillary Voltage: 3 kV; Desolvation gas (L/hr): 800; Polarity mode: Positive. Compound-specific mass spectrometric and chromatographic parameters for each compound are summarized in Supplementary Table S4. The details of the oxylipin chromatographic and MS conditions have been previously published^26^. For quantification purposes, a calibration curve was prepared for each lipid mediator platform by spiking 10 μl of the internal standard mixture to 8, 7 and 10 calibration points for sphingolipids, endocannabinoids and oxylipins, respectively. A calibration curve was injected every 24 samples and linear regression curves were built using the ratio between each compound and its respective internal standard applying a 1/x weighting. The ratio between each compound and its respective internal standard was then interpolated in the calibration curve.

### Statistical analysis

Statistical analysis was conducted using Stata Version 12 (StataCorp LP, College Station, TX, USA), Graph Pad Prism 5.0 for Windows (GraphPad Software, San Diego, CA, USA) and Matlab (Mathworks, Natick, MA, USA). Fischer’s exact test (for categorical variables) was used for the comparison of demographic and exposure variables between the groups in Table 1. For each compound, linear regression on each outcome adjusted for the other two as covariates was performed to assess the association between a variable and the compound. The Huber´s sandwich estimator^27^ for the standard errors of the regression coefficient was used to provide robustness to heteroscedasticity across covariate patterns. Q-values were calculated using the Storey method^28^. For compounds presenting a significant p-value and q-value for a given outcome (*i.e.,* gestational period), multiple comparisons with Bonferroni post hoc correction were performed. Spearman´s rho and statistical correlation significance were determined using Spearman´s rank correlation.

## Additional Information

**How to cite this article**: Checa, A. *et al.* Lipid mediator profile in vernix caseosa reflects skin barrier development. *Sci. Rep.* **5**, 15740; doi: 10.1038/srep15740 (2015).

## Supplementary Material

Supplementary Information

## Figures and Tables

**Table 1:** Demographic data of the VC samples analyzed in the study according to the different factors studied: gestational period, gender of the child and lifestyle of the mother.

Gestational period	Earlyterm	Full term	Post-term	p*
N	37	86	33	
Lifestyle (Anthro/Partly anthro/Non-anthro)	7/12/18	14/34/38	11/12/10	0.25
Gender of the child (female/male)	18/19	47/39	18/15	0.82
**Gender of the child**	**Female**	**Male**		p*
N	83	73		
Lifestyle (Anthro/ Partly anthro/Non-anthro)	13/32/38	19/26/28		0.27
Gestational period (Early/ Full /Post-term)	18/47/18	19/39/15		0.82
**Lifestyle**	**Anthro**	**Partly Anthro**	**Non-Anthro**	p*
N	32	58	66	
Gender of the child (female/male)	13/19	32/26	38/28	0.28
Gestational period (Early /Full /Post-term)	7/14/11	12/34/12	18/38/10	0.26

Gestational period was defined as early, full and post-term for children born between 36–38, 39–40 and 41–42 gestational weeks, respectively. Lifestyle is presented in terms of anthroposophic (Anthro), partly anthroposophic (Partly anthro) and non-anthroposophic lifestyle (Non anthro).

^*^p for comparison of the different categorical variables calculated using the χ^2^ test.

**Table 2:** Demographic data including differences in families with an anthroposophic, partly anthroposophic and non-anthroposophic lifestyle.

	Anthro	Partly Anthro	Non-Anthro	p*
N	32	58	66	
Pregnancy				
Mother				
Age at delivery	30.9 ± 6.8	30.8 ± 5.1	31.6 ± 4.1	0.68
Antibiotics	5/27 (18.5)	9/48 (18.6)	9/57 (15.8)	0.89
Vegetarian diet	9/32 (28.1)	10/58 (17.2)	1/62 (1.6)	**0.0002**
Organic/biodynamic diet	28/31 (90.3)	36/56 (64.3)	4/66 (6.1)	**1.7E-19**
Butter on bread	27/32 (84.4)	37/57 (64.9)	12/66 (18.2)	**1.3E-11**
Butter when cooking	15/31 (48.4)	19/57 (33.3)	10/64 (15.6)	**0.003**
Smoking	2/32 (6.3)	4/58 (6.9)	7/66 (10.6)	0.75
Delivery				
Home delivery	14/32 (43.8)	14/58 (24.1)	0/66 (0)	**6.6E-9**
Delivery mode				0.20
Normal delivery	27/32 (84.4)	52/58 (89.7)	50/66 (75.8)	
Vacuum extraction	1/32 (3.1)	3/58 (5.2)	1/66 (1.5)	
Acute caesarean	2/32 (6.3)	1/58 (1.7)	6/66 (9.1)	
Elective caesarean	2/32 (6.3)	2/58 (3.4)	9/66 (13.6)	

Bold indicates p < 0.05. Categorical variables: n/N (%). Continuous variables: mean ± SD.

^*^p for comparison of the lifestyle groups anthro. *vs.* partly anthro. *vs.* non-anthro. Categorical variables: p for trend (Fisher´s exact test); continuous variables: p for comparisons of means (one-way ANOVA).
