# Supplementary material for: Lipid mediator profile in vernix caseosa reflects skin barrier development
Source: Sci Rep. 2015 Nov 2;5:15740. doi: 10.1038/srep15740 (PMC4629127; doi:10.1038/srep15740)
Supplement: Supplementary Information [file srep15740-s1.pdf]

Supplementary material

Lipid mediator profile in vernix caseosa reflects skin barrier development

Antonio Checa[1], Tina Holm[2], Marcus O. D. Sjödin[1], Stacey N. Reinke[1], Johan Alm[3], Annika Scheynius[2], Craig E. Wheelock*[1]

[1] Division of Physiological Chemistry 2, Department of Medical Biochemistry and Biophysics, Karolinska Institutet, Stockholm, Sweden

[2] Translational Immunology Unit, Department of Medicine Solna, Karolinska Institutet and University Hospital, Stockholm, Sweden

[3] Department of Clinical Science and Education, Södersjukhuset, Karolinska Institutet and Sachs´ Children and Youth Hospital, Stockholm, Sweden

Corresponding author:

Craig E. Wheelock, PhD

Division of Physiological Chemistry II, Department of Medical Biochemistry and Biophysics

Karolinska Institutet

Scheeles väg 2, SE-171 77 Stockholm, Sweden

Phone number: +46 8 52487630; Fax:+46 8 736 04 39; e-mail: craig.wheelock@ki.se

Includes Supplementary Tables 1-4 and Supplementary Figure 1.

Supplementary Table 1. Lipid mediator concentrations of compounds found in vernix samples (n=156).

**Sphingolipids**						
**Compound**	**Mean[a]**	**Median[a]**	**Minimum[a]**	**Maximum[a]**	**% Samples[b]**	**% CV[c]**
Cer12:0	33.4	25.3	0.4	160.9	100.0	18.8
Cer14:0	1057	945	136	5279	100.0	26.2
Cer16:0	18643	17856	7499	56330	100.0	14.5
Cer18:0	4900	4728	2104	16259	100.0	13.3
Cer18:1	841	749	103	4496	100.0	16.7
Cer20:0	1894	1741	603	6209	100.0	11.5
Cer22:0	4866	4517	2486	12615	100.0	3.8
Cer24:0	18037	16804	9097	58766	100.0	8.3
Cer24:1	2699	2454	1017	7528	100.0	5.9
SM12:0	1000	880	137	7621	100.0	16.5
SM16:0	113699	108965	30788	237354	100.0	8.4
SM18:0	10296	9418	41	23366	100.0	8.1
SM18:1	5357	5056	40	14004	100.0	10.5
SM24:0	16068	15352	4949	38172	100.0	21.2
SM24:1	48022	47371	12160	114708	100.0	15.8
HexCer16:0	1290.1	1108.6	17.5	5483.2	100.0	20.1
HexCer18:0	209.5	177.0	32.5	897.0	100.0	20.0
HexCer18:1	37.1	36.5	0.4	92.4	100.0	14.8
HexCer24:1	1376.2	1147.1	274.6	7944.1	100.0	25.4
LacCer16:0	3937.2	2249.9	73.5	54698.2	100.0	20.0
LacCer24:0	945.2	658.4	177.8	15547.7	100.0	18.8
LacCer24:1	3701.6	1703.8	488.9	100068.5	100.0	15.0
DhCer16:0	4440.3	4102.3	1518.9	15858.9	100.0	7.9

Oxylipins

Compound	Mean[a]	Median[a]	Minimum[a]	Maximum[a]	% Samples[b]	% CV[c]
5-HETE	11.8	4.3	n.d.	320.5	88.5	6.4
9-HETE	337.0	135.0	11.9	9182.3	100.0	12.7
11-HETE	5.4	3.8	n.d.	68.7	89.7	11.0
15-HETE	30.6	20.3	3.4	576.1	100.0	25.0
5-KETE	2.3	0.8	n.d.	58.3	73.1	8.1
15-KETE	10.0	8.0	0.8	47.6	100.0	18.3
5-6-EpETrE	2.2	1.3	n.d	19.0	75.0	14.3
9-KODE	137.9	136.4	15.0	555.7	100.0	9.2
13-KODE	43.6	33.3	6.3	225.4	100.0	13.4
EKODE	9.9	8.2	2.9	91.0	100.0	26.5
9(10)-EpOME	3.0	2.0	0.2	20.6	100.0	27.9
12(13)-EpOME	4.1	2.0	0.2	24.6	100.0	21.0
9,10-DiHOME	4.3	3.0	0.2	33.5	100.0	29.1
12,13-DiHOME	7.6	4.6	1.3	36.8	100.0	10.8
5-HETrE	7.7	5.6	0.4	86.6	100.0	10.2
15-HETrE	5.6	3.4	n.d.	179.9	99.4	29.3
9-KOTrE	2.4	2.2	n.d.	7.4	82.1	13.5
12-HEPE	17.2	7.8	n.d.	335.7	97.4	14.5
11-HDoHE	13.4	7.6	n.d.	289.7	95.5	8.8
14-HDoHE	190.7	65.5	n.d.	10320.5	98.1	20.6
17-HDoHE	24.9	15.6	n.d.	657.3	99.4	26.8

Endocannabinoids and related ethanolamines

Compound	Mean[a]	Median[a]	Minimum[a]	Maximum[a]	% Samples[b]	% CV[c]
AEA	1.9	1.5	0.4	7.1	100.0	8.7
PEA	159.2	135.6	58.1	397.7	100.0	6.9
DihommoLEA	1.5	1.3	n.d.	4.4	98.7	11.3
LEA	19.3	15.6	4.1	83.2	100.0	14.9
OEA	29.4	24.7	1.7	77.4	100.0	14.9
1-AG	217.9	146.8	5.4	1583.9	100.0	12.8
2-AG	31.3	23.5	3.6	296.9	100.0	10.0
1-LG	634.1	488.9	54.6	4527.6	100.0	12.3
2-LG	179.8	80.7	10.7	5149.1	100.0	11.3
D(h)EA	5.2	4.6	1.3	15.4	100.0	12.5

Cer: Ceramide; SM: Sphingomyelin; DhCer: Dihydroceramide; Hexcer: Hexosylceramide; LacCer: Lactosylceramide; HETE: Hydroxy-eicosatetraenoic acid ; KETE: oxo-eicosatetraenoic acid; EpETre: epoxy-eicosatrienoic acid; KODE: oxo-octadecadienoic acid; EKODE: epoxy-keto-octadecenoic acid; EpOME: epoxy-octadecenoic acid; DiHOME: dihydroxy-octadecenoic acid; HETre: hydroxy-eicosatrienoic acid; KOTrE: oxo-octadecatrienoic acid; HEPE: hydroxy-eicosapentaenoic acid; HDoHE: hydroxy-docosahexaenoic acid; AEA: Arachidonoyl ethanolamide; PEA: Palmitoyl ethanolamide; LEA: Linoleoyl ethanolamide; OEA: Oleoyl ethanolamide; DIHOMOLEA: Dihomo-ϒ-linolenoyl ethanolamide; D(h)EA: Docosahexaenoyl ethanolamide; 1-AG: 1-arachidonoyl ethanolamide; 2-AG: 2-arachidonoyl ethanolamide; 1-LG: 1-linoleoyl ethanolamide; 2-LG: 2-linoleoyl ethanolamide

a) Amounts are expressed in ng of compound / g vernix.

b) Percentage of samples with compound above the limit of quantitation.

c) Coefficient of variance based on 6 replicates of a pooled vernix sample (see Materials and Methods for description).

Supplementary Table 2. Spearman´s correlations between the available ceramide to sphingomyelin ratios (Cer/SM) and the levels of the endocannabinoids anandamide (AEA) and 2-arachydonoyl glycerol (2-AG) in vernix caseosa samples (n=156).

**Compound**		**$Cer/SM_{12:0}$**	**$Cer/SM_{16:0}$**	**$Cer/SM_{18:1}$**	**$Cer/SM_{18:0}$**	**$Cer/SM_{24:1}$**	**$Cer/SM_{24:0}$**
**AEA**	ρ[a]	0.28	0.40	0.41	0.20	0.18	0.11
	p[b]	4.4 E-4	3.2 E-7	1.7 E-7	1.4 E-2	4.4 E-2	1.7 E-1
**2-AG**	ρ[a]	0.54	0.48	0.49	0.34	0.27	0.38
	p[b]	4.1 E-13	3.1 E-10	1.4 E-10	1.4 E-5	1.0 E-3	1.0 E-5

a) Spearman´s rank correlation

b) p-value

Supplementary Table 3. MS and chromatographic specific parameters for compounds included in the sphingolipid platform.

Compound	IS	RT (min)	Transition	Precursor ion	CV (V)	CE (V)
SM 12:0	SM 17:0	3.38	647.5/184.1	$[M+H]^+$	35	45
SM 16:0	SM 17:0	4.20	703.5/184.1	$[M+H]^+$	40	50
SM 18:1	SM 17:0	4.32	729.5/184.1	$[M+H]^+$	40	50
SM 18:0	SM 17:0	4.60	731.5/184.1	$[M+H]^+$	40	50
SM 24:1	SM 17:0	5.36	813.6/184.1	$[M+H]^+$	40	50
SM 24:0	SM 17:0	5.59	815.6/184.1	$[M+H]^+$	40	50
Cer 12:0	Cer 17:0	3.48	464.5/264.3	$[M+H-H2O]^+$	28	25
Cer 14:0	Cer 17:0	3.70	492.4/264.3	$[M+H-H2O]^+$	28	35
Cer 16:0	Cer 17:0	4.04	520.5/264.3	$[M+H-H2O]^+$	28	35
Cer 18:1	Cer 17:0	4.36	546.5/264.3	$[M+H-H2O]^+$	30	35
Cer 18:0	Cer 17:0	4.69	548.5/264.3	$[M+H-H2O]^+$	30	35
Cer 20:0	Cer[d17:1/24:1]	5.06	576.5/264.3	$[M+H-H2O]^+$	30	35
Cer 22:0	Cer[d17:1/24:1]	5.38	604.6/264.3	$[M+H-H2O]^+$	30	35
Cer 24:1	Cer[d17:1/24:1]	5.39	630.6/264.3	$[M+H-H2O]^+$	30	35
Cer 24:0	Cer[d17:1/24:1]	5.70	632.6/264.3	$[M+H-H2O]^+$	30	35
DhCer 16:0	DhCer6:0	4.39	540.5/266.4	$[M+H]^+$	30	35
HexCer 12:0	GlcCer8:0	3.26	626.5/264.4	$[M+H-H2O]^+$	35	40
HexCer 16:0	GlcCer8:0	4.06	682.5/264.4	$[M+H-H2O]^+$	35	40
HexCer 18:1	GlcCer8:0	4.19	708.5/264.4	$[M+H-H2O]^+$	35	40
HexCer 18:0	GlcCer8:0	4.46	710.5/264.4	$[M+H-H2O]^+$	35	40
HexCer 24:1	GlcCer8:0	5.22	792.6/264.4	$[M+H-H2O]^+$	35	40
LacCer 12:0	LacCer 17:0	3.16	788.6/264.4	$[M+H-H2O]^+$	45	50
LacCer 16:0	LacCer 17:0	3.95	844.6/264.4	$[M+H-H2O]^+$	35	40
LacCer 24:1	LacCer 17:0	5.12	954.7/264.4	$[M+H-H2O]^+$	40	60
LacCer 24:0	LacCer 17:0	5.42	958.8/264.4	$[M+H-H2O]^+$	50	65

SM: Sphingomyelin; Cer: Ceramide; DhCer: Dihydroceramide; Hexcer: Hexosylceramide; LacCer: Lactosylceramide; RT: Retention time; CV: Capillary Coltage; CE: Collision energy

Supplementary Table 4. MS and chromatographic specific parameters for compounds included in the endocannabinoid platform.

Compound	IS	RT (min)	Transition	Precursor ion	CV (V)	CE (V)
AEA	AEA-d4	5.35	348.2/62.0	$[M+H]^+$	20	20
PEA	PEA-d4	6.24	300.2/62.0	$[M+H]^+$	20	20
EPEA	LEA-d4	4.67	346.2/62.0	$[M+H]^+$	15	20
D(h)EA	D(h)EA-d4	5.18	372.2/62.0	$[M+H]^+$	15	20
LEA	LEA-d4	5.41	324.2/62.0	$[M+H]^+$	25	20
D(t)EA	OEA-d4	6.46	376.4/62.0	$[M+H]^+$	20	25
OEA	OEA-d4	6.54	326.2/62.0	$[M+H]^+$	25	20
SEA	OEA-d4	7.45	328.2/62.0	$[M+H]^+$	25	20
α-LINOLEA	LEA-d4	4.66	322.6/62.0	$[M+H]^+$	20	20
ϒ-DIHOMOLEA	LEA-d4	5.90	350.2/62.0	$[M+H]^+$	20	20
2-AG	2-AG-d5	5.85	379.2/287.2	$[M+H]^+$	20	15
2-LG	2-AG-d5	6.05	355.2/263.2	$[M+H]^+$	15	15

AEA: Arachidonoyl ethanolamide; PEA: Palmitoyl ethanolamide; EPEA: Eicosapentaenoyl ethanolamide; D(h)EA: Docosahexaenoyl ethanolamide; LEA: Linoleoyl ethanolamide; D(t)EA: Docosatetraenoyl ethanolamide; OEA: Oleoyl ethanolamide; SEA: Stearoyl ethanolamide; α-LINOLEA: α-linolenoyl ethanolamide; ϒ-DIHOMOLEA: Dihomo-ϒ-linolenoyl ethanolamide; 2-AG: 2-arachidonoyl ethanolamide; 2-LG: 2-linoleoyl ethanolamide; RT: Retention time; CV: Capillary Voltage; CE: Collision energy

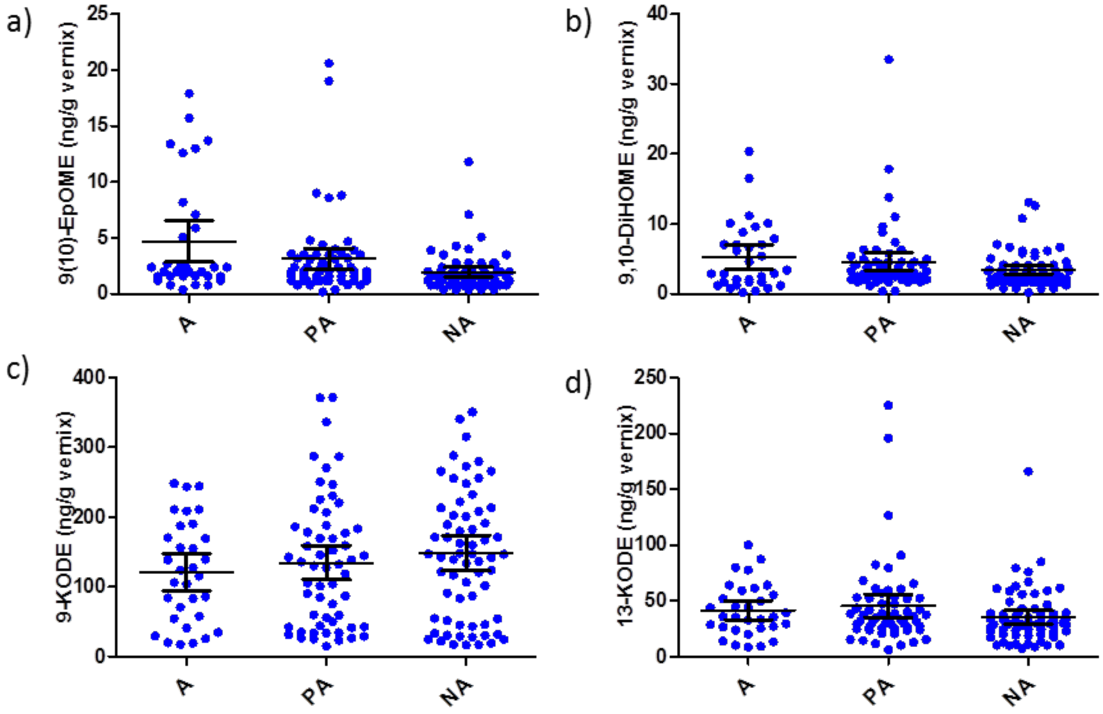

Supplementary Figure 1. Phenotypic differences in levels of a) 9(10)-EpOME, b) 9,10-DiHOME, c) 9-KODE and d) 13-KODE according to lifestyle (A = anthroposophic; P = partly anthroposophic; NA = non-anthroposophic). Each point represents an individual. The arithmetic mean with 95% confidence intervals is presented. None of the compounds was associated with lifestyle (see Figure 1). For graphical reasons one point is not presented on the NA group for 9-KODE (555 ng / g of vernix) group.
